# Electronic, Mechanical and Elastic Anisotropy Properties of X-Diamondyne (X = Si, Ge)

**DOI:** 10.3390/ma12213589

**Published:** 2019-10-31

**Authors:** Qingyang Fan, Zhongxing Duan, Yanxing Song, Wei Zhang, Qidong Zhang, Sining Yun

**Affiliations:** 1 College of Information and Control Engineering, Xi’an University of Architecture and Technology, Xi’an 710055, China; fanqy@xauat.edu.cn (Q.F.); zhx_duan@163.com (Z.D.); 2 School of Microelectronics, Xidian University, Xi’an 710071, China; syx739686768@163.com (Y.S.); zw_xidian@163.com (W.Z.); 3 Functional Materials Laboratory (FML), School of Materials Science and Engineering, Xi’an University of Architecture and Technology, Xi’an 710055, China

**Keywords:** group 14-diamondyne, mechanical anisotropy, direct band gap, thermal conductivity

## Abstract

The three-dimensional (3D) diamond-like semiconductor materials Si-diamondyne and Ge-diamondyne (also called SiC_4_ and GeC_4_) are studied utilizing density functional theory in this work, where the structural, elastic, electronic and mechanical anisotropy properties along with the minimum thermal conductivity are considered. SiC_4_ and GeC_4_ are semiconductor materials with direct band gaps and wide band gaps of 5.02 and 5.60 eV, respectively. The Debye temperatures of diamondyne, Si- and Ge-diamondyne are 422, 385 and 242 K, respectively, utilizing the empirical formula of the elastic modulus. Among these, Si-diamondyne has the largest mechanical anisotropy in the shear modulus and Young’s modulus, and Diamond has the smallest mechanical anisotropy in the Young’s modulus and shear modulus. The mechanical anisotropy in the Young’s modulus and shear modulus of Si-diamondyne is more than three times that of diamond as determined by the characterization of the ratio of the maximum value to the minimum value. The minimum thermal conductivity values of Si- and Ge-diamondyne are 0.727 and 0.524 W cm^−1^ K^−1^, respectively, and thus, Si- and Ge-diamondyne may be used in the thermoelectric industry.

## 1. Introduction

Carbon atoms have many ways of hybridizing in nature and can assume many allotropic forms [1,2,3,4,5,6,7,8,9,10,11,12,13,14,15,16,17,18,19,20]. Diamond is a typical *sp*^3^ hybrid product. It is a superhard and ultrawide band gap semiconductor material known in nature. Graphite is a typical *sp*^2^ hybridization product and is the most stable phase among the carbon isotopes. Graphite is also a conductor. Carbon allotropes consisting of *sp*-*sp*^2^ or *sp*^2^-*sp*^3^ hybrids can easily exhibit excellent physical properties, such as Dirac cones [21,22,23,24,25,26]. Diamondyne is also referred to as Y carbon [27] and 1-diamondyne [28], it inserts two carbon atoms between every two carbon atoms in the diamond structure. Therefore, silicides and germanides with *sp* or *sp*^2^ hybrid carbon should also have excellent physical properties. Recently, Sun et al. [29] designed a semiconductor material, namely SiC_4_, which is a wide-bandgap semiconductor with a high elasticity and low density. SiC_4_ has a wide band gap, good thermal stability, ultraviolet absorption of shading, low dark current and high photoelectric conversion efficiency. Its ultralight, ultraflexible and incompressible mechanical properties also enable photoelectric devices to meet various requirements in practical applications. This discovery prompted the study of silicides with carbon–carbon triple bonds (C≡C bond). Cao et al. [30] conducted A(X≡Y)_4_ (A = Si, Ge; X/Y = C, B, N) compound first-principles calculations, and they found that the A(X≡Y)_4_ (A = Si, Ge; X/Y = C, B, N) compounds have strong absorption in a wide ultraviolet range and exhibit supersoft, superlight and incompressible mechanical properties, and their optoelectronic and mechanical properties can be effectively adjusted by structural modification. The SiC_4_ and A(X≡Y)_4_ (A = Si, Ge; X/Y = C, B, N) compounds are all diamond-like structures. Very recently, a monocrystalline silicon-like material, C_40_H_16_Si_2_, Si(C≡C–C_6_H_4_–C≡C)_4_ was designed by Fang et al. [31]. The Si(C≡C–C_6_H_4_–C≡C)_4_ compound is a semiconductor material with a direct wide band gap, and its band gap is 3.32 eV. In addition, the Si(C≡C–C_6_H_4_–C≡C)_4_ compound is a low-density flexible porous material with strong absorption ability in the ultraviolet region. It is a promising semiconductor material for blue and green light-emitting diodes.

Using density functional theory [32,33], the physical properties of eight 3D diamond-like semiconductor materials, X-diamondyne (X = Si and Ge), diamond-Si, diamond-Ge, zinc blende-SiC, diamond-GeC, diamondyne and diamond, are investigated in this work. The minimum thermal conductivities of Si-diamondyne and Ge-diamondyne are very small; thus, Si_1−*x*_Ge*_x_*-diamondyne may be applied in the thermoelectric industry and perhaps could be used as a renewable energy device in green buildings, such as phase change materials [34].

## 2. Theoretical Methods 

The projects herein were carried out utilizing density functional theory within the ultrasoft pseudopotentials [35] method, as implemented in the Cambridge Sequential Total Energy Package (CASTEP). The exchange correlation potentials were adopted within the Perdew–Burke–Ernzerhof (PBE) functional of the generalized gradient approximation (GGA) [36]. The Heyd–Scuseria–Ernzerhof (HSE06) hybrid functional [37] was adopted for the calculations of the electronic band structures of X-diamondyne (X = Si and Ge), and the Broyden–Fletcher–Goldfarb–Shanno (BFGS) [38] minimization scheme was used for the geometric optimization of the X-diamondyne (X = Si and Ge). A high *k*-point separation (less than or approximately 0.025 Å^−1^) was used for X-diamondyne (X = Si and Ge), including 4 × 4 × 4 for the conventional cell and 6 × 6 × 6 for the primitive cells of the Si-diamondyne and Ge-diamondyne. For diamond, diamond-Si, diamond-Ge, zinc blende-SiC, diamond-GeC and diamondyne, 12 × 12 × 12, 8 × 8 × 8, 8 × 8 × 8, 10 × 10 × 10, 10 × 10 × 10 and 4 × 4 × 4 were used for the conventional cells, respectively. In addition, the *E*_cutoff_ energy of 400 eV was used for property prediction and structural optimization of the Si-diamondyne, Ge-diamondyne, zinc blende-SiC, diamond-GeC, diamondyne and diamond, a plane-wave cutoff energy of 340 eV was used for the diamond-Si, and the *E*_cutoff_ energy of 260 eV was used for the diamond-Ge. 

## 3. Results and Discussion

### 3.1. Structural Properties

**Figure 1:**
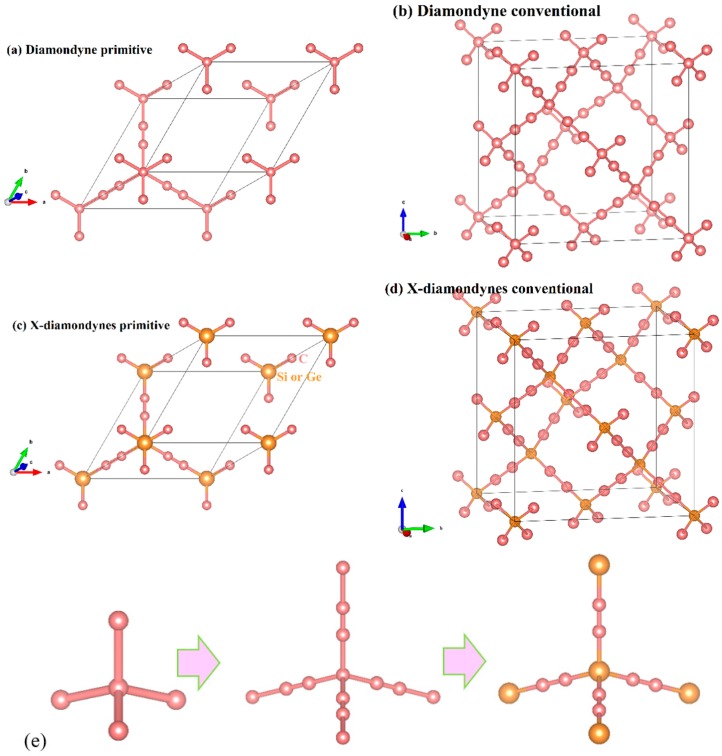
The crystal structures of (**a**,**b**) diamondyne; (**c**,**d**) Si-diamondyne; and (**e**) tetrahedral structures of diamond, diamondyne and Ge-diamondyne, from left to right.

**Figure 2:**
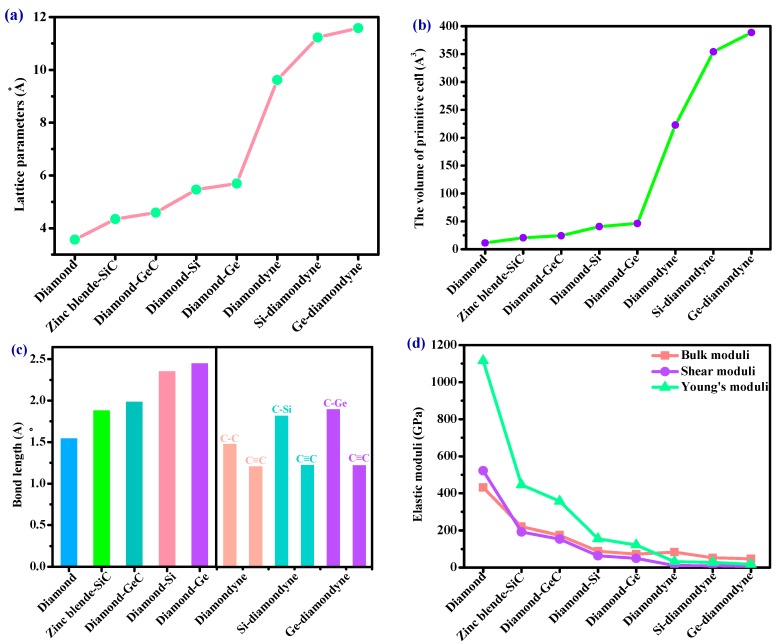
The (**a**) lattice parameters, (**b**) the volume of primitive cell, (**c**) bond lengths and (**d**) elastic moduli of diamond, zinc blende-SiC, diamond-GeC, diamond-Si, diamond-Ge, diamondyne, Si-diamondyne and Ge-diamondyne.

The crystal structures of the diamondyne and X-diamondyne (Si-diamondyne, SiC_4_; Ge-diamondyne, GeC_4_) are shown in Figure 1a–d. Because Si- and Ge-diamondyne and diamond, diamond-Si, diamond-Ge, zinc blende-SiC, diamond-GeC, and diamondyne have similar regular tetrahedral structures, many of the physical properties of Si- and Ge-diamondyne in this work are compared and discussed with them. For the diamondyne and diamond, the side length value of the regular tetrahedral structure of diamond is lengthened in the diamondyne, whereas the Si-diamondyne or Ge-diamondyne are formed by substituting silicon or germanium atoms for the central carbon atoms of the tetrahedral structure of diamond and diamondyne, as shown in Figure 1e. The calculated lattice constants of X-diamondyne, diamond-Si, diamond-Ge, zinc blende-SiC, diamond-GeC, diamondyne and diamond are shown in Table 1. The calculated theoretical lattice constants of diamond and zinc blende-SiC are both in good agreement with their experimental values as listed in Table 1. These theoretical results of diamond and zinc blende-SiC all support the physical properties of X-diamondyne (X = Si and Ge). The calculated lattice parameters all the materials studied in this work also increase in this order shown in Figure 2a. As seen in Figure 2a and Table 1, from diamond to diamond-Ge, the lattice constants increase by only 59.67%, while increasing by 103.44% from diamond-Ge to Ge-diamondyne. This is also reflected in the rate at which the primitive cell volume (i.e., the volume of their tetrahedral structure) increases, as shown in Table 1 and Figure 2b. The increase in the primitive cell volume is due to the increase of lattice constants on the one hand, and more importantly, to the increase of the atomic bond number on the other hand. The bond lengths of diamond, zinc blende-SiC, diamond-GeC, -Si and -Ge, diamondyne, and Si- and Ge-diamondyne are also shown in Figure 2c; it is clear that the increase in the cell volume is due to the increase of atomic bond length from diamond to diamond-Ge, while the increase in the cell volume is due to the increase of the atomic bond number from diamondyne to Ge-diamondyne. For Si- and Ge-diamondyne, the bond length of the C–C triple bond (C≡C) is almost unchanged, but only the Ge–C bond increases more than that of the Si–C bond.

### 3.2. Electronic Properties

**Figure 3:**
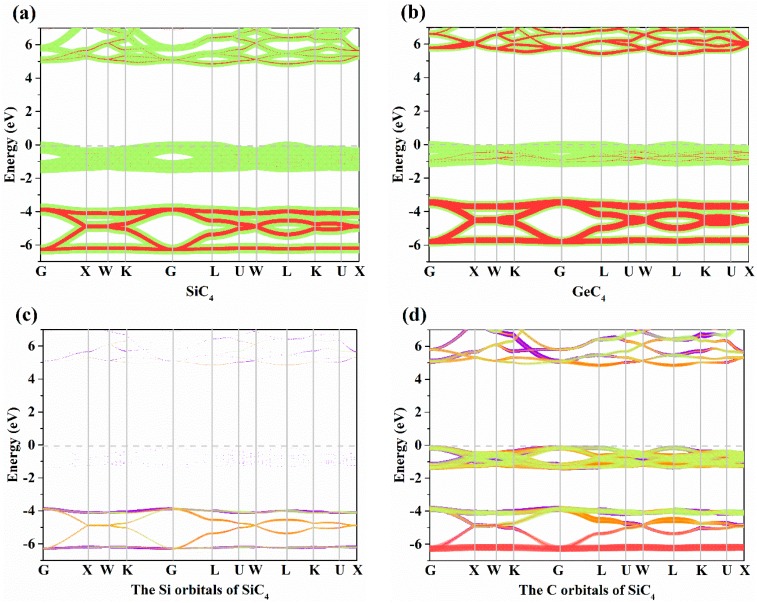

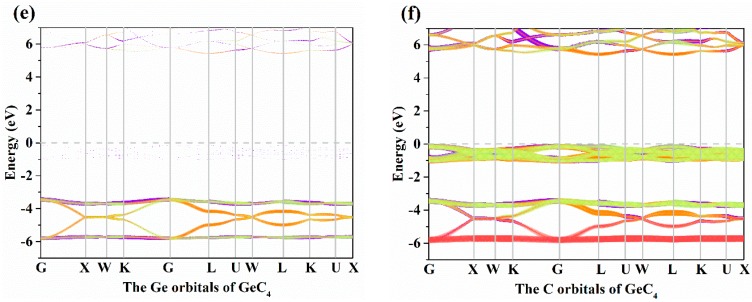
The electronic band structures for (**a**) Si-diamondyne and (**b**) Ge-diamondyne; the electronic band structures for (**c**) Si atom and (**d**) C atom of Si-diamondyne; and the electronic band structures for (**e**) Ge atom and (**f**) C atom of Ge-diamondyne.

The electronic band structures of X-diamondyne (X = Si and Ge) with a HSE06 hybrid functional are displayed in Figure 3a,b. Here, the sizes of the red and green circles represent the projected weight of the *s* and *p* orbitals, respectively, of Si or Ge atoms and C atoms. For Si- and Ge-diamondyne, both the SiC_4_ and GeC_4_ are direct band gap and wide semiconductor materials, where the band gaps of SiC_4_ and GeC_4_ are 5.02 and 5.60 eV, respectively, within the HSE06 hybrid functional. The X-diamondyne are wide band gap semiconductor materials. The band gaps of SiC_4_ and GeC_4_ are in good agreement with the reported theoretical values in [29] (5.02 eV) and [30] (5.59 eV). The band gaps of SiC_4_ and GeC_4_ are 3.80 and 4.27 eV, respectively, within the PBE functional and the band gap of GeC_4_ is in excellent agreement with the theoretical value (4.38 eV) in [30]. From Figure 3a,b, the C *p* electrons and *s* electrons provide a major contribution to the Fermi level and valence-band maximum (VBM) for X-diamondyne, and the *p* electrons and *s* electrons for Si or Ge contribute less to the Fermi level and VBM. For SiC_4_ and GeC_4_, the contributions of the *p* electrons and *s* electrons in GeC_4_ are greater than those in SiC_4_. The electronic band structures of different atoms in X-diamondyne are plotted in Figure 3c–f. Here, the size of the red, orange, violet and green circles illustrate the projected weight of the *s*, *p_x_*, *p_y_* and *p_z_* orbitals, respectively. Figure 3c,e show that both the *s*, *p_x_*, *p_y_* and *p_z_* orbitals of the silicon or germanium atoms mentioned above contribute to a low degree to the Fermi level and VBM. The electrons in the C atoms, namely the *p_z_* electrons of X-diamondyne, strongly contribute to the Fermi level, and the *p_x_* electrons of X-diamondyne strongly contribute to the VBM. The coordinates of high symmetry points in the Brillouin zone for SiC_4_ and GeC_4_ are G (0.000, 0.000, 0.000) → X (0.500, 0.000, 0.500) → W (0.500, 0.250, 0.750) → K (0.375, 0.375, 0.375) → G (0.000, 0.000, 0.000) → L (0.500, 0.500, 0.500) → U (0.625, 0.250, 0.750) → W (0.500, 0.250, 0.750) → L (0.500, 0.500, 0.500) → K (0.375, 0.375, 0.375) → U (0.625, 0.250, 0.750) → X (0.500, 0.000, 0.500). The VBM and the CBM of X-diamondyne are located at the L point, and the coordinate of the high symmetry point of L is the same as the R point in [29].

### 3.3. Elastic Properties and Mechanical Anisotropy Properties

Elastic modulus is a momentous performance parameter of engineering materials. From the macroscopic point of view, the elastic modulus is an index to measure the ability of an object to resist elastic deformation. From a microscopic point of view, it reflects the bonding strength between atoms, ions or molecules. The calculated elastic moduli and elastic constants in Table 1 are very close to the theoretical and experimental values reported previously. They both decrease with the substitution of atoms in the regular tetrahedral structure. When carbon atoms are replaced by silicon atoms, the *C*_11_ of the zinc blende-SiC is 62.96% less than that of diamond, and the *C*_44_ is 55.42% less than that of diamond. When the carbon atoms with regular tetrahedral structures are completely replaced by silicon atoms, the *C*_11_ of diamond-Si is 85.38% less than that of diamond, and the *C*_44_ is 85.97% less than that of diamond. However, when the side length of the tetrahedron increases in length—that is to say, after increasing the C≡C bond—the *C*_11_ of diamondyne decreases by 91.45% compared to that of diamond, and the *C*_44_ decreases by 96.63%. The elastic moduli of all the materials studied in this work are illustrated in Figure 2d, including the shear moduli, bulk moduli, and Young’s moduli. Among the materials considered herein, the bulk modulus, shear modulus and Young’s modulus of diamond are the greatest, and those of Ge-diamondyne are the smallest. The calculated elastic moduli from greatest to least are in the following order: diamond > zinc blende-SiC > diamond-GeC > diamond-Si > diamond-Ge > diamondyne > Si-diamondyne > Ge-diamondyne. 

The Debye temperature is another momentous physical quantity reflecting the bonding force between atoms. The Debye temperature of the different materials is distinctive, and the melting point is high. That is, the higher the Debye temperature, the stronger the bonding force. The Debye temperature can be estimated by the empirical formula for the elastic modulus. The Debye temperature can be expressed by [48,49] *Θ*_D_ = (*h*/*k_B_*)[3*n*/(4π)(*N_A_ρ*/*M*)]^1/3^*v_m_*, where *v_m_* = [(2/$v_{s}^{3}$ + 1/$v_{p}^{3}$)/3]^−1/3^, *v_p_* = [(*B* + 4*G*/3)/*ρ*]^1/2^, *v_s_* = (*G*/*ρ*)^1/2^, *h* is Planck’s constant, *k_B_* is Boltzmann’s constant, *N_A_* is Avogadro’s number, *n* is the number of atoms in the molecule, *M* is the molecular weight, *ρ* is the crystal density, *v_p_* is the compressional sound wave velocity, *v_s_* is the shear sound wave velocity and *v_m_* is the mean sound velocity. The calculated compressional sound wave velocities, shear sound wave velocities, mean sound velocities and Debye temperature are listed in Table 2. Among the materials studied herein, the mean sound velocity of diamond is still the largest and that of diamond-Ge is the smallest because the crystal density of diamond-Ge is large, and the elastic moduli of diamond-Ge are small. When the silicon atom does not completely replace the carbon atom in the tetrahedron, the Debye temperature of the zinc blende-SiC decreases by 47.70% compared with that of diamond. The Debye temperature of the diamond-Si decreases by 71.26% when the silicon atom completely replaces the carbon atom in tetrahedron. After increasing the carbon–carbon triple bond (C≡C bond), the side length of the tetrahedron increases, but the Debye temperature of the diamondyne decreases by 80.99% compared to that of diamond.

**Figure 4:**
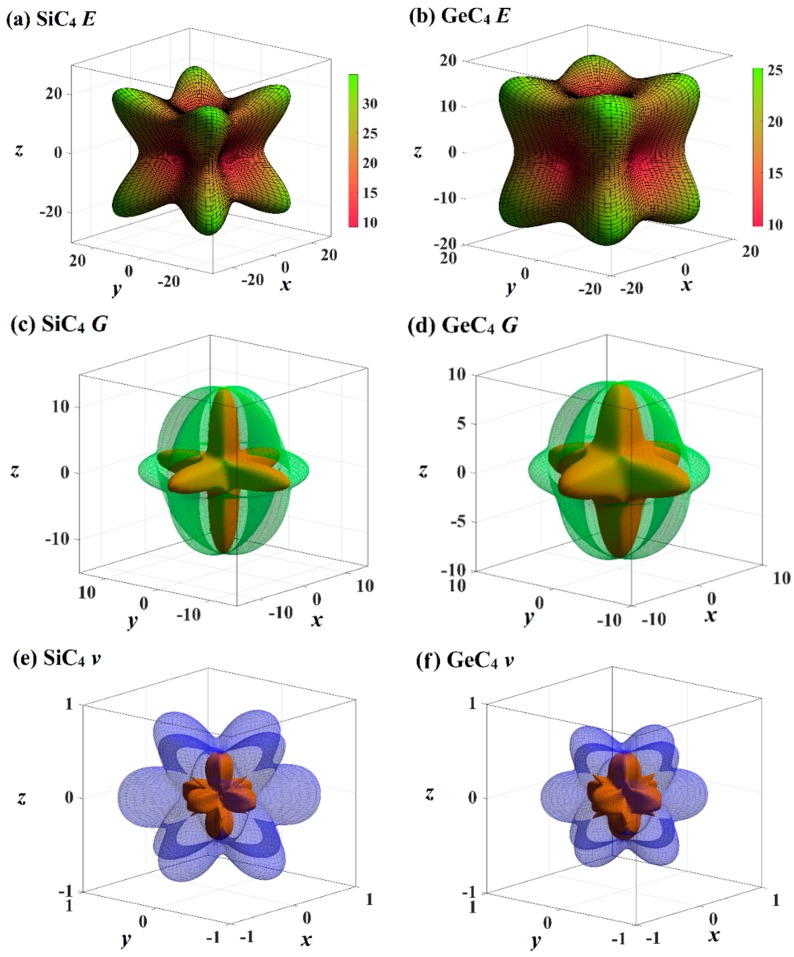
The three-dimensional contour plots of Young’s modulus for (**a**) Si-diamondyne and (**b**) Ge-diamondyne; the three-dimensional contour plots of shear modulus for (**c**) Si-diamondyne and (**d**) Ge-diamondyne; and the three-dimensional contour plots of Poisson’s ratio for (**e**) Si-diamondyne and (**f**) Ge-diamondyne.

**Figure 5:**
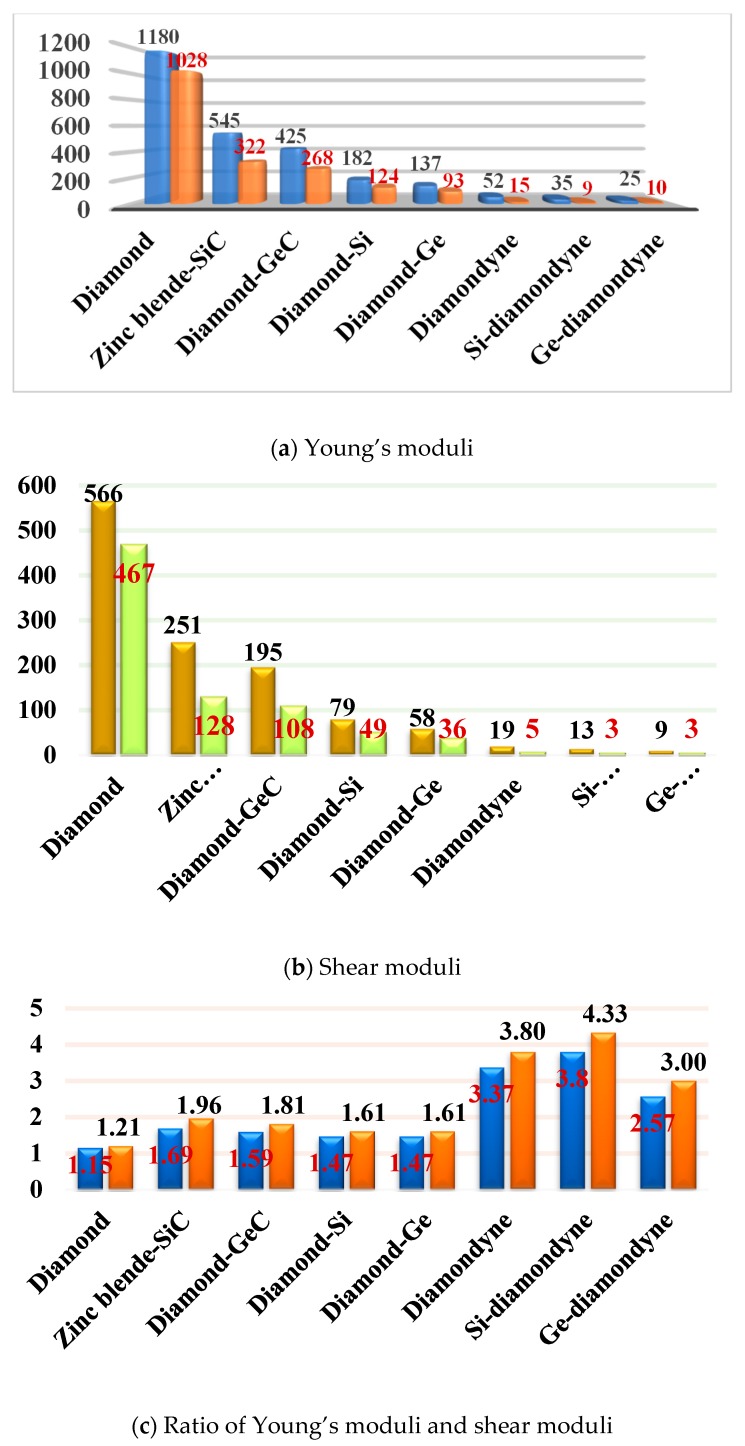
(**a**) The maximum values and the minimum values of Young’s modulus; (**b**) the maximum values and the minimum value of shear modulus; and (**c**) the *E*_max_/*E*_min_ and *G*_max_/*G*_min_ ratios of diamond, zinc blende-SiC, diamond-GeC, diamond-Si, diamond-Ge, diamondyne, Si-diamondyne and Ge-diamondyne.

The mechanical anisotropy can intuitively tell us in which direction the maximum value of a physical quantity appears and in which direction the minimum value appears. The 3D surface constructions of the shear modulus *G*, Young’s modulus *E*, and Poisson’s ratio for Si- and Ge-diamondyne are shown in Figure 4. If the material is elastic isotropic, the three-dimensional view of its elastic modulus is a sphere [51,52,53]. For the Young’s moduli in Figure 4a,b, the Si- and Ge-diamondyne both exhibit mechanical anisotropy and the Si-diamondyne shows a larger mechanical anisotropy in Young’s modulus than the Ge-diamondyne. To explain this situation, we calculated the maximum and minimum values of the Young’s modulus and shear modulus for all the materials studied in this work. The results for the Young’s moduli and shear moduli are illustrated in Figure 5a,b. Among them, light blue and light orange represent the maximum and minimum values, respectively. As shown in Table 1, among the materials studied herein, the maximum value of the Young’s modulus for Diamond is still the greatest, while Si-diamondyne has the smallest maximum value of the Young’s modulus. For Si- and Ge-diamondyne, the maximum values of *E* are 35 GPa and 25 GPa, respectively, and the minimum values of Young’s modulus are 9 GPa and 10 GPa, respectively. The ratios of the maximum value to minimum value of Young’s modulus for all the materials are shown in Figure 5c, and the blue represents the *E*_max_/*E*_min_ ratio. From Figure 5c, it is clear that the Si-diamondyne has the greatest *E*_max_/*E*_min_ ratio among the materials; in other words, the Si-diamondyne has the largest mechanical anisotropy in *E* among them and diamond has the smallest mechanical anisotropy in *E*. The calculated mechanical anisotropy in the Young’s modulus from greatest to least are in the following order: Si-diamondyne > diamondyne > Ge-diamondyne > zinc blende-SiC > diamond-GeC > diamond-Si = diamond-Ge > Diamond.

To better understand the mechanical anisotropy of the Young’s modulus, we studied the distribution of the Young’s modulus in the main planes (such as (001), (010), (100), (101), (110), (111) and (011)). The ratios of the maximum to minimum value of the Young’s modulus in these planes are listed in Table 3. Because all the materials in this work have cubic symmetry, the distribution of the Young’s modulus in some of their planes is the same; that is, they have the same maximum and minimum values, such as in the (100), (010) and (001) planes and the (011), (101) and (110) planes. As shown in Table 3, the (111) plane of all the materials exhibits a mechanical isotropy in Young’s modulus. In addition, the (011), (101) and (110) planes of all the materials have a larger mechanical anisotropy than the (100), (010) and (001) planes.

The 3D surface constructions of the minimum value and the maximum value of shear modulus *G* for Si- and Ge-diamondyne are shown in Figure 4c,d, and the 3D surface constructions of the minimum value and the maximum value of Poisson’s ratio for Si- and Ge-diamondyne are shown in Figure 4e,f. Here, the green surface and the red surface represent the minimum value and the maximum value for *G*, respectively, and the violet surface and the red surface represent the maximum value and the minimum value for the shear modulus, respectively. From the three-dimensional view, we can also see that the shear modulus of the Si-diamondyne has a greater mechanical anisotropy than the Ge-diamondyne. For the shear modulus, the maximum and minimum values of the Young’s modulus and shear modulus for all the materials are shown in Figure 5b. Among the materials studied herein, the maximum value of *G* for diamond is still the greatest, while Si- and Ge-diamondyne both have the smallest maximum value of *G*. The maximum values and minimum values of the Poisson’s ratio and shear modulus are listed in Table 4. Among the materials studied herein, the maximum value of Poisson’s ratio for Si-diamondyne is the greatest, and the zinc blende-SiC, diamond-GeC, diamondyne and Si-diamondyne have the smallest Poisson’s ratio; the smallest Poisson’s ratio is zero. The ratios of the maximum value and minimum value of *G* are shown in Figure 5c where orange represents the *G*_max_/*G*_min_ ratio. As shown in Figure 5c and Table 4, Si-diamondyne has the largest mechanical anisotropy in the shear modulus, and diamond has the smallest mechanical anisotropy among the materials studied herein. The calculated mechanical anisotropies in the shear modulus from greatest to least are as follows: Si-diamondyne > diamondyne > Ge-diamondyne > zinc blende-SiC > diamond-GeC > diamond-Si = diamond-Ge > diamond. For the shear modulus and Young’s modulus, materials with carbon–carbon triple bonds (C≡C bond) exhibit greater mechanical anisotropy than those without carbon–carbon triple bonds.

### 3.4. The Minimum Thermal Conductivity

The theoretical estimation of the thermal conductivity is a hot topic in physical chemistry and condensed matter physics. Utilizing the compressional and shear sound wave velocities, the relation between the minimum thermal conductivity *κ*_min_ and temperature as expressed by Cahill et al. is as follows [54]:

(1) $$\kappa_{min}={\left(\frac{\pi }{6}\right)}^{1/3}k_{B}n^{2/3}\sum_{i}v_{i}{\left(\frac{T}{\Theta_{i}}\right)}^{2}\int_{0}^{\Theta_{i}/T}\frac{x^{3}e^{x}}{{\left(e^{x}-1\right)}^{2}}dx.$$

**Figure 6:**
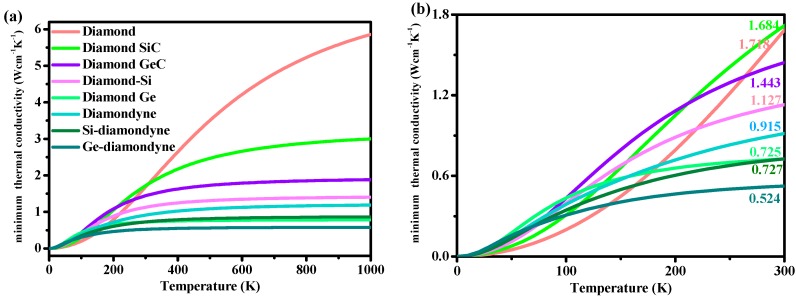
The relationships between temperature and the minimum thermal conductivity *κ*_min_ of diamond, zinc blende-SiC, diamond-GeC, diamond-Si, diamond-Ge, diamondyne, Si-diamondyne and Ge-diamondyne, (**a**) 0–1000 K; (**b**) 0–300 K.

Here, *v*_i_ is the compressional or shear sound wave velocity, *Θ_i_* is the cut-off frequency for each polarization expressed in K, *Θ_i_* = *v_i_*[*h*/(2π*k*_B_)](6π^2^*n*)^1/3^ and *T* is the temperature. This empirical formula has been used to predict the thermal conductivity of various materials [55,56,57]. The relations between the minimum thermal conductivity *κ*_min_ and the temperature (from 0 to 1000 K) of all the materials studied in this work are illustrated in Figure 6. As shown in Figure 6, with increasing temperature, the *κ*_min_ of diamond increases rapidly in the high-temperature region (400 K < *T* < 1000 K) compared with that in the low-temperature region (0 K < *T* < 400 K). As shown in Figure 6b, the minimum thermal conductivity of diamond is the smallest—under *T* < ~150 K—among the materials studied herein. When the temperature exceeds 150 K, the *κ*_min_ of diamond begins to exceed that of other materials. The zinc blende-SiC has the highest *κ*_min_ between 200 and 300 K among the materials studied herein, while Si- and Ge-diamondyne have the lowest thermal conductivity between 150 and 300 K. The calculated *κ*_min_ values under ambient temperature (300 K) are listed in Table 2, and they are also marked in Figure 6b. The minimum thermal conductivities of Si-diamondyne and Ge-diamondyne are 0.727 and 0.524 W cm^−1^ K^−1^, respectively, while the minimum thermal conductivity of Si-diamondyne is smaller than that of *t*-Si_64_ (0.74 W cm^−1^ K^−1^) [58]. The lower the thermal conductivity *κ* is, the greater the thermoelectric figure of merit *ZT* [58]. Therefore, it can be concluded that Si- and Ge-diamondyne may be applied in the thermoelectric industry.

## 4. Conclusions

Using density functional theory, the electronic properties, elastic properties, structural properties, mechanical anisotropy properties and *κ*_min_ of Si-diamondyne (SiC_4_) and Ge-diamondyne (GeC_4_) were investigated in this work. The lattice parameters of diamond-Ge increased by 103.44% compared to those of Ge-diamondyne; this increase was larger than that for diamond to diamond-Ge (only 59.67%). The electronic structures show that SiC_4_ and GeC_4_ are semiconductor materials with direct band gaps and wide band gaps of 5.02 and 5.60 eV, respectively, within the HSE06 hybrid functional. By displaying the three-dimensional graph and comparing the ratios of the maximum value to the minimum value, Si-diamondyne was shown to have the largest mechanical anisotropy in terms of both Young’s modulus and shear modulus, and diamond has the smallest mechanical anisotropy in terms of Young’s modulus and shear modulus among the materials studied herein. The calculated mechanical anisotropy in Young’s modulus and shear modulus from greatest to least was as follows: Si-diamondyne > diamondyne > Ge-diamondyne > Zinc blende-SiC > diamond-GeC > diamond-Si = diamond-Ge > diamond. The minimum thermal conductivities of Si-diamondyne and Ge-diamondyne were 0.727 and 0.524 W cm^−1^ K^−1^, respectively. Therefore, it can be concluded that Si-diamondyne and Ge-diamondyne may be applied in the thermoelectric industry.

## Figures and Tables

**Table 1:** The lattice parameters *a* (Å), density *ρ* (g/cm^3^), volume of primitive cell *V* (Å^3^), elastic constants (GPa), and elastic moduli (GPa) of diamond, zinc blende-SiC, diamond-GeC, diamond-Si, Diamond-Ge, diamondyne, Si-diamondyne and Ge-diamondyne.

	*a*	*ρ*	*V*	*C*_11_	*C*_12_	*C*_44_	*B*	*G*	*E*	*v*
Diamond	3.566	3.518	11.340	1053	120	563	431	522	1116	0.07
	3.567 ^a^			1076 ^b^	125	577	442			
Zinc blende-SiC	4.348	3.240	20.550	390	134	251	220	192	446	0.16
	4.360 ^c^			390 ^d^	142	256	227 ^e^			
Diamond-Si	5.464, 5.465 ^f^	2.288	40.773	154	56	79	88	64	155	0.21
	5.430 ^g^			165	64	87				
Diamond-GeC	4.590	5.811	24.175	318	102	195	174	154	357	0.16
	4.590 ^h^						175 ^i^			
Diamond-Ge	5.694	5.224	46.152	121	49	62	73	50	122	0.22
	5.660 ^g^	5.318	45.330	129	48	67	77			
Diamondyne	9.621	0.896	222.65	90	79	19	83	11	32	0.44
	9.628 ^j^	0.894	223.10				95			
	9.636 ^k^	0.892	223.68				83			
Si-diamondyne	11.233	0.713	354.36	58	51	13	53	10	28	0.41
	11.220 ^l^	0.740	353.12	59	54	14	55	7	20	0.48
Ge-diamondyne	11.584	1.031	388.64	51	45	9	47	6	17	0.44
	11.590 ^m^	1.030	389.22	55	48	7	50	5	15	0.48

^a^ [39]; ^b^ [40]; ^c^ [41]-experiment; ^d^ [42]; ^e^ [43]; ^f^ [44]; ^g^ [45]; ^h^ [46]; ^i^ [47]; ^j^ [28]; ^k^ [27]; ^l^ [29]; ^m^ [30].

**Table 2:** The compressional, shear and mean elastic wave velocity (*v_s_*, *v_p_*, *v_m_* in m/s), and the Debye temperature (*Θ*_D_ in K) of diamond, zinc blende-SiC, diamond-GeC, diamond-Si, diamond-Ge, diamondyne, Si-diamondyne and Ge-diamondyne.

	Diamond	Zinc Blende-SiC	Diamond-GeC	Diamond-Si	Diamond-Ge	Diamondyne	Si-Diamondyne	Ge-Diamondyne
*v_p_*	17,898	12,121	8080	8704	5171	10,440	9645	7304
*v_s_*	12,181	7698	5148	5289	3094	3504	3745	2412
*v_m_*	13,280	8465	5659	5843	3423	3986	4246	3745
*Θ*_D_	2220	1161, 1232 ^a^	734	638	358	422	385	242
*κ*_min_	1.684	1.718	1.443	1.127	0.725	0.915	0.727	0.524

^a^ [50].

**Table 3:** The maximum values and the minimum values of Young’s modulus (in GPa) and *E*_max_/*E*_min_ in primary planes for diamond, zinc blende-SiC, diamond-GeC, diamond-Si, diamond-Ge, diamondyne, Si-diamondyne and Ge-diamondyne.

	(001) (010) (100)			(011) (101) (110)			(111)			All		
	*E*_max_	*E*_min_	Ratio	*E*_max_	*E*_min_	Ratio	*E*_max_	*E*_min_	Ratio	*E*_max_	*E*_min_	Ratio
Diamond	1138	1028	1.11	1180	1028	1.15	1138	1138	1.00	1180	1028	1.15
Zinc blende-SiC	465	322	1.44	545	322	1.69	465	465	1.00	545	322	1.69
Diamond-GeC	371	268	1.38	425	268	1.59	371	371	1.00	425	268	1.59
Diamond-Si	163	124	1.31	182	124	1.47	163	163	1.00	182	124	1.47
Diamond-Ge	123	93	1.32	137	93	1.47	123	123	1.00	137	93	1.47
Diamondyne	33	15	2.20	52	15	3.47	33	33	1.00	52	15	3.47
Si-diamondyne	21	9	2.33	35	9	3.89	21	21	1.00	35	9	3.89
Ge-diamondyne	18	10	1.80	25	10	2.50	18	18	1.00	25	10	2.50

**Table 4:** The maximum values and the minimum values of Poisson’s ratio, shear modulus (in GPa) and *G*_max_/*G*_min_ in primary planes for diamond, zinc blende-SiC, diamond-GeC, diamond-Si, diamond-Ge, diamondyne, Si-diamondyne and Ge-diamondyne.

	*V*		*G*		
	*v*_max_	*v*_min_	*G*_max_	*G*_min_	*G*_max_/*G*_min_
Diamond	0.11	0.01	566	467	1.21
Zinc blende-SiC	0.37	0.00	251	128	1.96
Diamond-GeC	0.34	0.00	195	108	1.81
Diamond-Si	0.35	0.04	79	49	1.61
Diamond-Ge	0.38	0.06	58	36	1.61
Diamondyne	0.99	0.00	19	5	3.80
Si-diamondyne	1.06	0.00	13	3	4.33
Ge-diamondyne	0.86	0.01	9	3	3.00
